# Vesicular stomatitis virus with the rabies virus glycoprotein directs retrograde transsynaptic transport among neurons *in vivo*

**DOI:** 10.3389/fncir.2013.00011

**Published:** 2013-02-07

**Authors:** Kevin T. Beier, Arpiar B. Saunders, Ian A. Oldenburg, Bernardo L. Sabatini, Constance L. Cepko

**Affiliations:** ^1^Department of Genetics and Department of Ophthalmology, Harvard Medical School, Harvard University and Howard Hughes Medical InstituteBoston, MA, USA; ^2^Department of Neurobiology, Harvard Medical School, Harvard University and Howard Hughes Medical InstituteBoston, MA, USA

**Keywords:** vesicular stomatitis virus, transsynaptic infection, rabies, retrograde transneuronal tracing, *in vivo*, technology, polysynaptic

## Abstract

Defining the connections among neurons is critical to our understanding of the structure and function of the nervous system. Recombinant viruses engineered to transmit across synapses provide a powerful approach for the dissection of neuronal circuitry *in vivo*. We recently demonstrated that recombinant vesicular stomatitis virus (VSV) can be endowed with anterograde or retrograde transsynaptic tracing ability by providing the virus with different glycoproteins. Here we extend the characterization of the transmission and gene expression of recombinant VSV (rVSV) with the rabies virus glycoprotein (RABV-G), and provide examples of its activity relative to the anterograde transsynaptic tracer form of rVSV. rVSV with RABV-G was found to drive strong expression of transgenes and to spread rapidly from neuron to neuron in only a retrograde manner. Depending upon how the RABV-G was delivered, VSV served as a polysynaptic or monosynaptic tracer, or was able to define projections through axonal uptake and retrograde transport. In animals co-infected with rVSV in its anterograde form, rVSV with RABV-G could be used to begin to characterize the similarities and differences in connections to different areas. rVSV with RABV-G provides a flexible, rapid, and versatile tracing tool that complements the previously described VSV-based anterograde transsynaptic tracer.

## Introduction

Mapping neuronal connectivity in the central nervous system (CNS) of even simple organisms is a difficult task. Recombinant viruses engineered to trace synaptic connections and express transgenes promise to enable higher-throughput mapping of connections among neurons than other methods, e.g., serial reconstruction from electron micrographs (Bock et al., 2011; Briggman et al., 2011). The Pseudorabies (PRV) and Rabies viruses (RABV) have been the best characterized and most utilized circuit tracing viruses to date (Ugolini et al., 1989; Kelly and Strick, 2000). RABV was recently modified by Wickersham and colleagues such that it can travel across only one synapse, allowing for a straightforward definition of monosynaptic connections (Wickersham et al., 2007b). This strategy permitted the first unambiguous identification of retrogradely connected cells from an initially infected cell (“starter cell”), without the need for electrophysiology. Moreover, the starter cell could be defined through the expression of a specific viral receptor that limited the initial infection.

**Figure 1:**
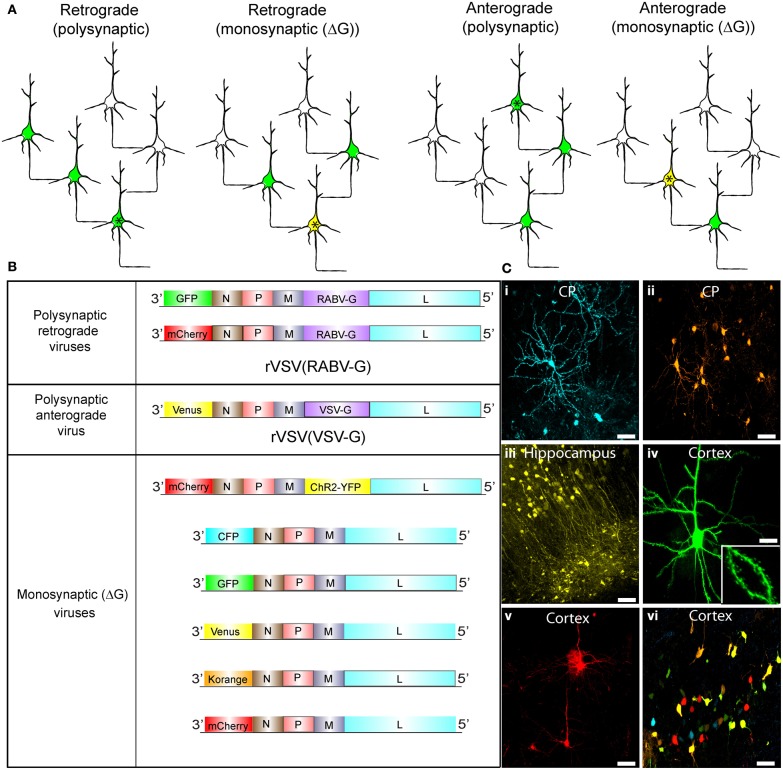
**Synaptic tracing strategies using VSV. (A)** Schematic illustrating the strategies for polysynaptic or monosynaptic retrograde or anterograde transsynaptic transmission of rVSV encoding GFP. The initially infected cell is indicated by an asterisk. VSV encoding a glycoprotein (G) within its genome can spread polysynaptically. The direction of the spread depends on the identity of the glycoprotein. Infected neurons are shown in green. In some cases, the initially infected starter cell can be defined by the expression of an avian receptor, TVA (tagged with a red fluorescent protein). The TVA-expressing neurons can then be specifically infected by rVSVΔG with the EnvA/RABV-G (A/RG) glycoprotein (Wickersham et al., 2007b) on the virion surface [rVSVΔG(A/RG)]. These starter cells are then yellow, due to viral GFP and mCherry from TVA-mCherry expression. For monosynaptic tracing, the G protein is expressed *in trans* in the TVA-expressing cell, and thus complements rVSVΔG to allow transmission in a specific direction. **(B)** Genomic diagrams of rVSV vectors. All VSVs contain four essential proteins: N, P, M, and L. Some viruses encode a G gene in their genome, which allows them to spread polysynaptically. rVSV vectors typically encode a transgene in the first position, while others carry an additional transgene in the G position. **(C)** Morphological characterization of rVSV-infected neurons in several locations within the mouse brain. **(i,ii)** Caudate-putamen (CP) neurons at 4 dpi from an injection of the CP with rVSV(VSV-G) viruses encoding **(i)** CFP or **(ii)** Korange. **(iii)** Labeled neurons of the CA1 region of the hippocampus are shown at 5 dpi following injection into the hippocampus of rVSV(VSV-G) encoding Venus. **(iv,v)** Cortical pyramidal neurons are shown following injection into the CP of rVSV(RABV-G) expressing **(iv)** GFP at 24 hpi, or **(v)** mCherry at 48 hpi. Inset in **(iv)** is a high magnification of the neuron in panel **(iv)**, highlighting labeling of dendritic spines. **(vi)** Multiple viruses can be co-injected into the same animal. Here, individual rVSVΔG(VSV-G) viruses encoding CFP, GFP, Venus, Korange, and mCherry were used to infect the cortex. Scale bars = 50 μm.

Recently, we created an anterograde monosynaptic virus that complements the previously available retrograde viral tracers (Beier et al., 2011). Vesicular stomatitis virus (VSV), a virus related to RABV, with its own glycoprotein (G) gene (VSV-G), or with a G from the unrelated lymphocytic choriomeningitis virus (LCMV-G), spreads in the anterograde direction across synapses. VSV can be used as a polysynaptic tracer that spreads across many synapses, owing to the fact that the normal, replication-competent form of the virus does not cause serious diseases in humans (Brandly and Hanson, 1957; Johnson et al., 1966; Brody et al., 1967). Whether the virus is a monosynaptic or polysynaptic tracer is determined by the method of delivery of the G gene (Figure 1A). Advantages of VSV are that it is well-characterized, is relatively simple in comparison to PRV, and it rapidly grows to high titer in tissue culture cells. It is also being developed as a vaccine vector, often using a G of another virus as the immunogen, as well as being developed as a cytocidal agent that will target tumor cells in humans (Balachandran and Barber, 2000; Stojdl et al., 2000, 2003).

Previous studies of the anatomical patterns of transmission, as well as physiological recordings, have shown that the transmission of VSV and RABV among neurons is via synapses (Kelly and Strick, 2000; Wickersham et al., 2007b; Beier et al., 2011). In addition, it has been shown that RABV, as well as lentiviruses with RABV-G in their envelope, travel retrogradely from an injection site (Mazarakis et al., 2001; Wickersham et al., 2007a). We hypothesized that providing a recombinant VSV (rVSV) with the RABV-G would create a retrograde polysynaptic transsynaptic tracer without the biosafety concerns inherent to RABV. Our initial characterization of rVSV with RABV-G showed that indeed it could be taken up as a retrograde tracer (Beier et al., 2011). To determine if it could transmit among neurons following its replication in neurons, and to further analyze the transmission patterns of both the monosynaptic and polysynaptic forms of rVSV with RABV-G, we made injections into several CNS and peripheral locations. In addition, we performed co-infections of rVSV with RABV-G and the anterograde form of rVSV in order to exploit the differences in the directionality of transmission of these two viruses in mapping circuits.

## Results

### VSV can encode a variety of transgenes

Schematics of viruses created and used throughout this study are shown in Figure 1. We created rVSV vector plasmids carrying different transgenes in either the first or fifth genomic positions (Figure 1B). After rescuing each virus, we tested the ability of each to express transgenes in different brain regions through intracranial injections (Figure 1C). All rVSV vectors drove robust fluorophore expression 1 or 2 days post-infection (hpi) (Figure 1C) (van den Pol et al., 2009). In fact, by 12 hpi, labeling was sufficiently bright to image fine morphological details, such as dendritic spines (Figure 1C,**iv**).

### Physiology of cells infected with rVSV encoding RABV-G

**Figure 2:**
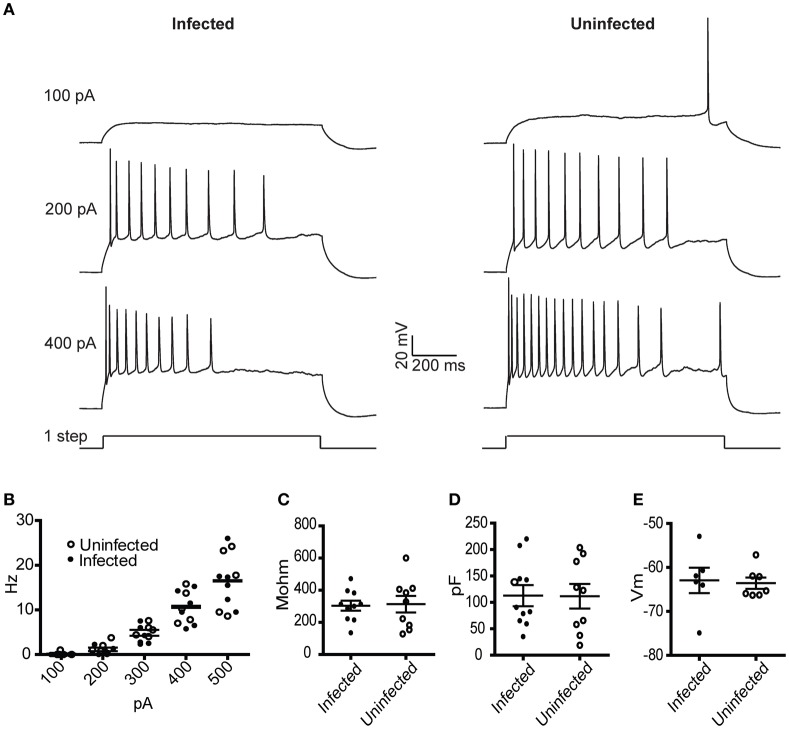
**Physiological characterization of rVSV-infected and uninfected layer 5 cortical pyramidal neurons following injection of rVSV(RABV-G) into the CP.** Slices were cut 12 hpi and recordings were taken over the subsequent 6 h. **(A)** Example spike trains driven by 100, 200, and 400 pA square current pulses lasting 1 s for infected (left) and uninfected (right) neurons. **(B)** A summary plot showing current/action potential firing frequency relationships are unaffected by infection (infected cells, *N* = 7; uninfected cells, *N* = 6). Horizontal bars denote averages. Infection does not alter the **(C)** input resistance, the **(D)** capacitance, or **(E)** resting membrane voltages (infected cells, *N* = 10, uninfected cells, *N* = 9). Horizontal bars denote mean with standard error of the mean.

To characterize the physiological properties of cells infected with rVSV, we tested a replication-competent rVSV encoding GFP, with RABV-G in the genome in place of VSV-G [hereafter designated rVSV(RABV-G)]. van den Pol et al. reported that hippocampal neurons infected with replication-incompetent (G-deleted or “ΔG”) rVSV were physiologically healthy at 12–14 hpi, but were less so by 1 day post-infection (dpi) (van den Pol et al., 2009). Given the known toxicity of both VSV and RABV-G (Coulon et al., 1982), we tested the physiology of cortical pyramidal neurons in the motor cortex (M1) infected with rVSV(RABV-G). Between 12 and 18 hpi, the membrane capacitance, input resistance, resting membrane potential, and current-to-action potential firing relationship were indistinguishable between infected and uninfected neurons (Figure 2). However, by 2 dpi, electrophysiological properties were so abnormal in the infected cortical pyramidal cells that physiological measurements could not be made.

### VSV expresses transgenes rapidly in neurons

The speed and strength of the expression of transgenes encoded by VSV depends upon the gene's genomic position (van den Pol et al., 2009; Beier et al., 2011). Genes in the first position are expressed the most highly, with a decrease in the level of expression in positions more 3′ within the viral plus strand. When GFP was inserted into the first position of VSV, GFP fluorescence was first detectable at approximately 1 hpi in cultured cells (van den Pol et al., 2009).

**Figure 3:**
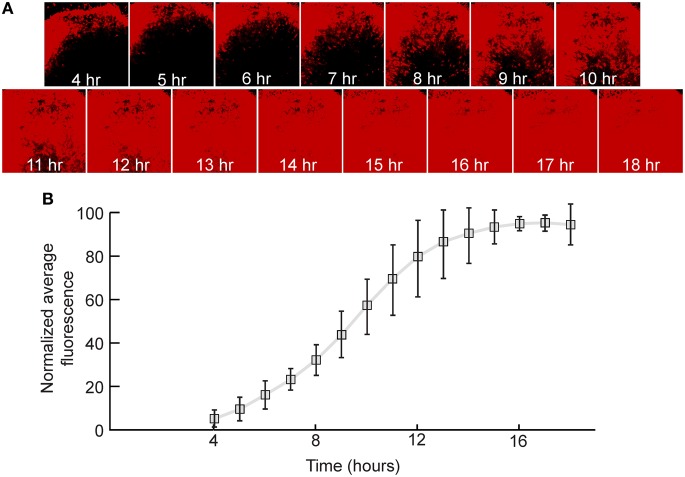
**Quantification of viral transgene expression over time. (A)** After 7 days *in vitro* (DIV), hippocampal slice cultures were infected with rVSVΔG(RABV-G) expressing mCherry in the first genomic position. Sample images of the same visual field are shown over time. **(B)** Fluorescence was quantified as a function of time and was normalized to expression at 18 hpi. The plot indicates averages ± 1 SD, *N* = 3.

In order to quantify the relative expression of a fluorescent protein in the first genomic position in neurons, rat hippocampal slices were infected with a replication-incompetent rVSV that expresses mCherry (rVSVΔG, Figures 1A,B). This was a ΔG virus which had the RABV-G supplied in trans during the preparation of the virus stock [referred to as rVSVΔG(RABV-G)]. Average fluorescence intensity of the infected cells was measured every hour over the course of 18 h. By 4 hpi at 37°C, red fluorescence was clearly visible, and reached maximal levels by approximately 14 hpi (*N* = 3, Figure 3). Similar results were obtained with a virus encoding GFP in the first genomic position rather than mCherry (i.e., Figure 1B) (*N* = 3).

### rVSV(RABV-G) spreads transsynaptically in the retrograde direction

**Figure 4:**
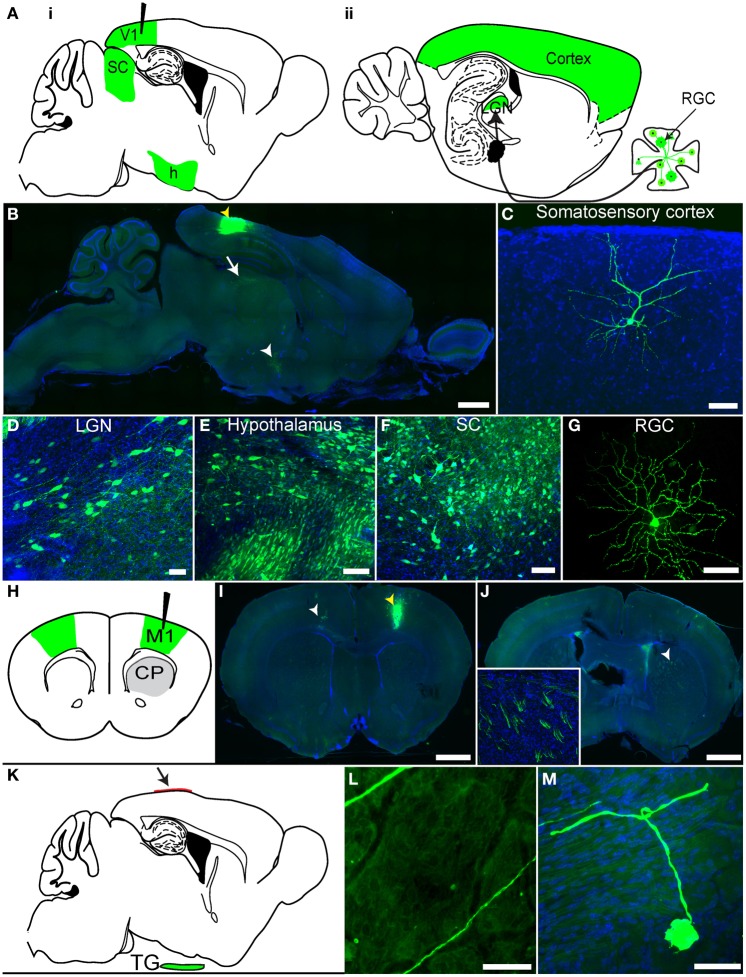
**rVSV(RABV-G) exhibits polysynaptic retrograde spread *in vivo*. (A)** Schematics of two parasaggital sections separated by 1.3 mm are shown. rVSV(RABV-G) injected into V1 (black needle) should yield infected cells in the labeled areas shown in green, including RGCs in the retina (panel **ii**). Areas projecting directly to V1, such as the hypothalamus (h), LGN, as well as other cortical areas, can be labeled by direct retrograde uptake of injected virions, whereas RGCs, which project to the LGN, can only be labeled by secondary viral spread. **(B)** rVSV(RABV-G) was injected into V1 (yellow arrowhead), and both the brain and retina examined 7 dpi. Infection in the brain appeared to be primarily in directly projecting areas, including the surrounding cortices, the LGN (white arrow), and hypothalamus (white arrowhead). Higher magnifications of labeled cells from a V1 injection are shown in panels **(C–G)**. **(C)** somatosensory cortex, 7 dpi; **(D)** LGN, 3 dpi; **(E)** hypothalamus, 3 dpi; **(F)** SC, 3 dpi; **(G)** RGC, 3 dpi. **(H)** Schematic of a coronal section showing rVSV(RABV-G) injected into M1 (black needle). The contralateral cortex (green) should be labeled by this virus, while at early time points such as 2 dpi, the CP, which receives projections from the cortex but does not itself send projections to the cortex, should not (gray). **(I)** Coronal section showing GFP-labeled neurons in M1, imaged 4 dpi. The injection site was in M1, indicated by a yellow arrowhead, with neurons projecting to the injection site indicated by the white arrowhead. **(J)** CP neuronal cell bodies were not labeled, but labeled cortical axon bundles running through the CP were observed (inset shows axon bundles in the area demarcated by the white arrowhead). **(K–M)** rVSV(RABV-G) can trace circuits into the CNS from a peripheral site. **(K)** Parasaggital schematic showing a predicted area of infection following infection of the dura with a retrogradely transported virus. rVSV(RABV-G) was applied to the intact dura (arrow) and if retrograde uptake and transport can occur, trigeminal ganglion neurons that project to the dura (green) should become labeled. **(L)** Examples of axons located on the dura, 3 dpi. Infected neuronal cell bodies were not located on the dura, **(M)** but instead were observed in the trigeminal ganglion. No infection of the brain was observed in these animals. Scale bars: **(B,I,J)** = 1 mm, **(L)** = 100 μm, **(C–G,M)** = 50 μm.

We previously demonstrated that rVSV(RABV-G) could be taken up retrogradely by neurons (Beier et al., 2011), but these experiments did not distinguish between direct axonal uptake of the initial inoculum vs. retrograde transsynaptic transmission following viral replication. To distinguish between these two mechanisms and to extend the previous analyses, we conducted further experiments in the mammalian visual system (Figures 4A–G). As visual cortex area 1 (V1) does not receive direct projections from retinal ganglion cells (RGCs), but rather receives secondary input from RGCs via the lateral geniculate nucleus (LGN), infection of RGCs from injection of V1 would demonstrate retrograde transmission from cells which supported at least one round of viral replication. Following a V1 injection with rVSV(RABV-G), GFP-positive RGCs were observed in the retina by 3 dpi (*N* = 3; Figure 4G). Importantly, viral labeling in the brain was restricted to primary and secondary projection areas, even at 7 dpi. These included the LGN (Figure 4D) and the hypothalamus (Figure 4E), two areas known to project directly to V1 (Kandel, 2000). Selective labeling was observed in other areas, such as cortical areas surrounding V1 (Figure 4C), which project directly to V1, and also in the superior colliculus (SC) stratum griseum centrale, which projects to the LGN (Figure 4F). Labeling was also observed in the nucleus basalis, which projects to the cortex, as well as many components of the basal ganglia circuit, which provide input to the thalamus [such as the caudate-putamen (CP), globus pallidus (GP), and the subthalamic nucleus (STn)]. The amygdala, which projects to the hypothalamus, was also labeled. Consistent with a lack of widespread viral transmission, animals did not exhibit signs of disease at 7 dpi.

These data show that rVSV(RABV-G) can spread in a retrograde direction from the injection site, but do not address whether the virus can spread exclusively in the retrograde direction. Directional transsynaptic specificity can only be definitively addressed using a unidirectional circuit. We therefore turned to the primary motor cortex (M1) to CP connection, in which neurons project from the cortex to the CP, but not in the other direction (Figure 4H) (Beier et al., 2011). Injections of rVSV(RABV-G) into M1 should not label neurons in the CP if the virus can only label cells across synapses in the retrograde direction. Indeed, at 2 dpi, areas directly projecting to the injection site, including the contralateral cortex, were labeled (Figure 4I). Only axons from cortical cells were observed in the CP, with no GFP-labeled cell bodies present in the CP (Figure 4J), consistent with lack of anterograde transsynaptic spread. By 3 dpi, a small number of medium spiny neurons (MSNs) in the CP were observed, likely via secondary spread from initially infected thalamic or GP neurons (data not shown).

### Peripheral uptake of rVSV(RABV-G) and transmission to the CNS

A particular advantage of retrograde viral tracers is the ability to label CNS neurons projecting to peripheral sites. This has been a powerful application of both RABV and PRV (Ugolini et al., 1989; Standish et al., 1994). To test if rVSV(RABV-G) could also perform this function, we examined the innervation of the dura surface by neurons of the trigeminal ganglion, a neuronal circuit thought to be involved in migraine headaches (Penfield and McNaughton, 1940; Mayberg et al., 1984). These neurons have axons, but not canonical dendrites, and send projections into the spinal cord and brainstem. Therefore, the only way trigeminal neurons could become labeled from viral application to the dura is through retrograde uptake of the virus.

We applied rVSV(RABV-G) to the intact dura mater and analyzed the dura, trigeminal ganglion, and CNS for labeling (Figure 4K). At the earliest time point examined, 3 dpi, we observed axons traveling along the dura, but little other evidence of infection (Figure 4L). No labeled neuronal cell bodies on the dura were observed, consistent with the lack of neurons on this surface. In contrast, we did find labeled cell bodies in the trigeminal ganglion (Figure 4M). No infection was seen in the CNS, even at 4 dpi, consistent with the lack of inputs from the brain into the trigeminal ganglion (*N* = 4 animals).

### The kinetics of retrograde transsynaptic spread

**Figure 5:**
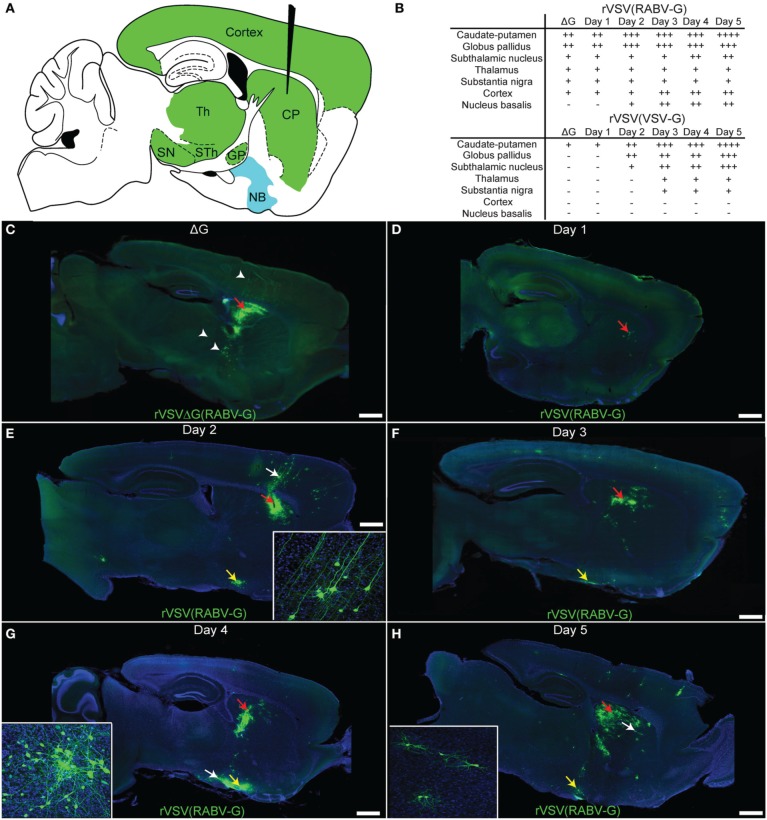
**Time course of rVSV(RABV-G) spread from the CP recapitulates the connectivity of known basal ganglia-thalamo-cortical circuits. (A)** A parasaggital schematic showing the relevant projections into and from the injection site in the CP. Black needle points to injection site, green = primary projecting regions, blue = secondary projecting region. CP, caudate-putamen; GP, globus pallidus; SN, substantia nigra; STh, subthalamic nucleus; Th, thalamus; NB, nucleus basalis. **(B)** Assessment of viral spread from rVSV(RABV-G) and rVSV(VSV-G) injections into the CP. The presence or absence of labeling is indicated by (+) and (−), respectively. The extent of labeling is indicated by the number of (+). Some animals were infected with ΔG viruses to determine which areas were labeled by direct uptake of the virions, rather than by replication and transmission. These were sacrificed at 3 dpi. **(C)** Parasaggital section of a brain infected with VSV[greek delta]G(RABV-G). The injection site is marked by a red arrow. Several areas that project directly to the CP were labeled due to direct uptake of the virions, including the cortex, thalamus, and GP (arrowheads), 3 dpi. **(D–H)** Replication-competent rVSV(RABV-G) was injected into the CP (red arrows), and the time course of labeling was monitored for 5 days [**(D)** 1 day, **(E)** 2, **(F)** 3, **(G)** 4, and **(H)** 5 days]. Insets show high magnifications of areas indicated by white arrows. Sections from animals at 1 dpi show labeling consistent with the initial infection [compare to rVSVΔG(RABV-G), panel **C**], while spread to secondarily connected areas, such as the nucleus basalis, was observed at 2 dpi (yellow arrows). Viral spread was relatively restricted to the basal ganglia circuit, even out to 5 dpi. Scale bars = 1 mm.

To further characterize patterns and kinetics of viral transmission and directional specificity of transsynaptic spread, injections of rVSV(RABV-G) were made into the CP (Figure 5A). In order to determine which cells were labeled by direct uptake of virus in the inoculum, a separate set of animals were injected into the CP with the replication-incompetent rVSVΔG(RABV-G) (*N* = 3 animals, analyzed 3 dpi). Cells labeled by rVSVΔG(RABV-G) were observed in the CP, GP, substantia nigra (SN), thalamus, and layers 3 and 5 of the cortex, consistent with infection at the axon terminal and retrograde labeling of cell bodies of neurons known to project directly to the CP (Figure 5C) (Albin et al., 1995). Areas labeled by CP injection are indicated in Figure 5B.

The patterns of spread for the replication-competent rVSV(RABV-G) were characterized over the course of 1–5 dpi (Figures 5D–H). During this interval, progressively more cells in infected regions were labeled by rVSV(RABV-G), including within the CP, nucleus basalis, cortex, and GP (listed in Figure 5B). In addition, more cortical cells were labeled in clusters near cortical pyramidal neurons, both ipsilateral and contralateral to the injected side, including neurogliaform cells (data not shown). These data are in contrast to those observed following infection with an anterograde transsynaptic tracing virus, such as rVSV with its own G gene, rVSV(VSV-G) (Figure 5B). At 3 dpi following rVSV(VSV-G) injection into the CP, the cerebral cortex was not labeled, but regions receiving projections from the CP, such as the STn, GP, and SN, were labeled (Beier et al., 2011).

In order to investigate other areas for evidence of cell-to-cell retrograde transsynaptic spread, the nucleus basalis was examined following infection of the CP with replication-competent rVSV(RABV-G). The nucleus basalis was labeled by 2 dpi (Figures 5E–H), consistent with at least a single transsynaptic jump, as this area does not directly project to the CP. The virus appeared to travel transsynaptically at the rate of roughly 1 synapse per day, as evidenced by the lack of labeled neurogliaform cells in the cortex, and lack of neurons in the nucleus basalis at 1 dpi, and label appearing in these cell types/areas at 2 dpi, as previously observed (Beier et al., 2011). Labeling remained well-restricted to the expected corticostriatal circuits at 5 dpi, suggesting that viral spread becomes less efficient after crossing one or two connections, consistent with injections into V1 (Figure 4). While glial cells can be infected and were observed near the injection site (van den Pol et al., 2002; Chauhan et al., 2010), infected glial cells away from the injection site generally were not observed.

### Polysynaptic tracers can be combined *in vivo*

**Figure 6:**
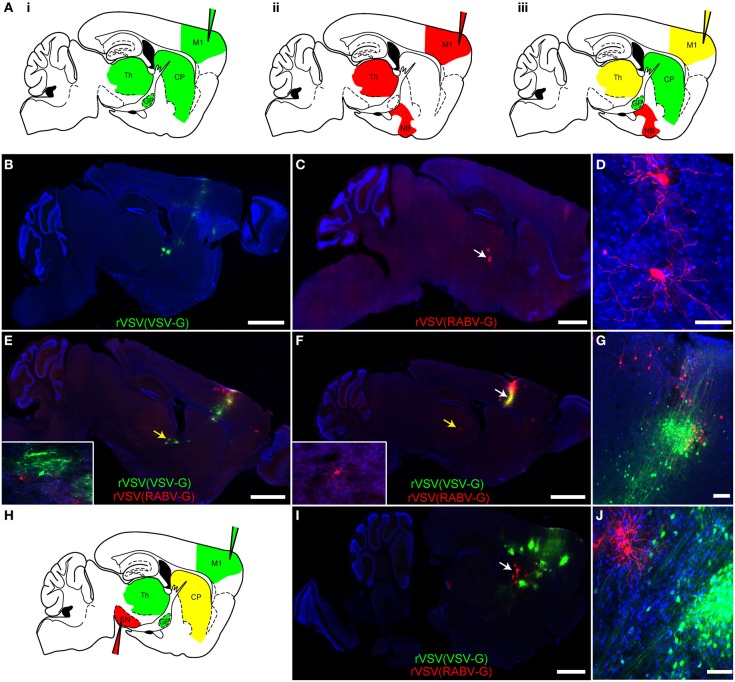
**Simultaneous anterograde and retrograde transsynaptic circuit tracing using rVSV. (A)** Connectivity schematics of parasaggital sections indicating patterns of spread from injection of M1 (injection needles) with a polysynaptic virus transmitting across synapses in the **(i)** anterograde or **(ii)** retrograde directions. Panel **(iii)** shows the pattern from co-injection of two polysynaptic viruses, one anterograde and one retrograde. Green represents the anterograde virus, red the retrograde virus, and yellow, both. Note that yellow indicates that the area is predicted to host infection by both viruses, with potentially some individual cells showing infection with both viruses. **(B)** The anterograde transsynaptic virus rVSV(VSV-G), when injected alone, labeled M1 as well as anterograde projection areas, such as the CP, GP, and thalamus, whereas **(C)** the retrograde virus rVSV(RABV-G) labeled M1 as well as areas projecting to the cortex, including the thalamus. **(D)** High magnification of thalamic cells shown in **(C)** (white arrow). **(E,F)** Examples taken from a series of parasaggital sections from the same brain of an animal injected with both viruses simultaneously into M1. Co-infection of cells in M1 was not observed, **(G)**, and no spurious labeling of anterograde or retrograde projection regions was observed—i.e., the combination of viruses was equal to the sum of each virus injected individually. Insets show high magnifications of thalamic neurons in **(E)** and **(F)** labeled by the two viruses (indicated by the yellow arrows) demonstrating no co-labeling. **(G)** A high magnification view of the injection site in the cortex shown in panel **(F)** (white arrow), showing independent labeling of neurons by each virus. **(H)** A schematic of a parasaggital section depicting the pattern of transmission of an anterograde (green) and retrograde (red) virus injected into two different areas of the basal ganglia circuit. This strategy can be used to connect multiple elements in a circuit. The rVSV(VSV-G) that expressed Venus (labeled cells depicted in green) was injected into M1, while the rVSV(RABV-G) that expressed mCherry was injected into the SN, where it labeled direct pathway MSNs in the CP (yellow). **(I)** Using these coordinates, largely non-overlapping regions of the CP were labeled by these viruses, as shown in **(J)**. Scale bars: **(B,C,E,F,I)** = 1 mm; **(D,G,J)** = 50 μm.

One advantage of having both anterograde and retrograde forms of the same virus is that they can be used in parallel, or in tandem, to trace circuitry to and from a single or multiple sites of injection, with each virus having similar kinetics of spread and gene expression. In fact, if different fluorophores are used in different viruses, e.g., rVSV(VSV-G) and rVSV(RABV-G), then the viruses can be co-injected into the same site and their transmission can be traced independently (Figure 6A). This is most straightforward if there are no cells at the injection site that are initially infected by both viruses. Co-infected cells can be easily detected, as they would express both fluorescent proteins shortly after injection.

In order to determine whether two viruses would allow simultaneous anterograde and retrograde transsynaptic tracing from a single injection site, a rVSV(VSV-G) expressing Venus and a rVSV(RABV-G) expressing mCherry were injected individually (Figures 6B–D) or co-injected (Figures 6E–G) into the motor cortex, and brains were examined 3 dpi. The pattern of labeling from the co-injected brains was equivalent to the patterns observed when each virus was injected individually: rVSV(VSV-G) was observed to infect neurons in the cortex, CP, and downstream nuclei, whereas the rVSV(RABV-G) was not observed to infect neurons in the CP, but rather in the thalamus and nucleus basalis (*N* = 4). The initial co-infection rate is dependent upon the dose of the initial inocula. When injecting 3 × 10^3^ focus forming units (ffu) rVSV(VSV-G) and 3 × 10^4^ ffu rVSV(RABV-G), no co-infection was observed at the injection site. Thus, co-infection of the same brain region, without co-infection of the same cells, does not alter the spreading behavior of either rVSV(VSV-G) or rVSV(RABV-G).

One example of how this dual retrograde and anterograde transsynaptic tracing system can be used is to determine if three distinct regions are connected and the directionality of any connections. For example, the anterograde transsynaptic virus can be injected into one region, the retrograde into another, and a third region can then be examined for evidence of labeling by either or both viruses (e.g., Figure 6H). To test this possibility, rVSV(VSV-G) was injected into the motor cortex, rVSV(RABV-G) was injected into the substantia nigra pars reticulara (SNr), and animals were sacrificed at 3 dpi. We observed that cells were singly labeled, either with Venus [rVSV(VSV-G)] or with mCherry [rVSV(RABV-G)], and were located largely in different regions of the CP (Figures 6I,J) (*N* = 3). These results suggest that the anterograde connections from the cells infected with rVSV(VSV-G) in the M1 were with CP MSNs that did not project to the region of the SNr injected with rVSV(RABV-G) (*N* = 3 animals).

### VSV can trace monosynaptically connected circuits in the retrograde direction

**Figure 7:**
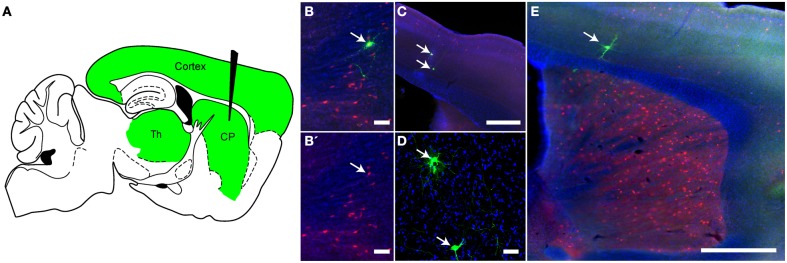
**Monosynaptic retrograde tracing using rVSV *in vivo*. (A)** A schematic of a parasaggital section showing the predicted pattern of monosynaptic retrograde spread from Choline Acetyltransferase (ChAT)-expressing neurons in the CP to directly connected cells. A combination of two Cre-dependent adeno-associated viruses (AAVs), one expressing a TVA-mCherry fusion protein and the other RABV-G, were injected into the CP of ChAT-Cre/Ai9 animals. This permits expression of the transgenes encoded in the AAVs in cells with a ChAT expression history. Two weeks later, rVSVΔG(A/RG), a G-deleted virus that only infects TVA-expressing neurons, was injected into the same region, and the brain was observed 5 days later. The injection of rVSV into the CP (black needle) should result in infection of TVA-expressing neurons in the CP. From these starter cells, monosynaptic spread could occur only to directly connected inputs such as those in the cortex and thalamus (green). (**B,B′**) Initially infected cells in the CP were both red (TVA-expressing) and green (rVSV infected) (arrow). **B′** shows the red and blue channels only, blue = DAPI. (**C–E**) Examples of rVSV-infected cells in the cortex **(C,E)** and thalamus **(D)** that were infected by monosynaptic transmission from the starter cells, (arrows indicate cells infected by transmission), *N* = 3. Scale bars: **(B,D)** = 50 μm, **(C,E)** = 500 μm.

In addition to polysynaptic tracing, VSV can be modified to trace circuits monosynaptically (Beier et al., 2011). With RABV, this was achieved *in vivo* by first infecting with an adeno-associated virus (AAV) expressing TVA, a receptor for an avian retrovirus, and RABV-G (Wall et al., 2010). This was followed 3 weeks later by infection with a ΔG RABV with an EnvA/RABV-G chimeric glycoprotein on the virion surface (Wickersham et al., 2007b), which allowed infection specifically of the cells expressing TVA. A similar strategy was used to test rVSV's ability to monosynaptically trace retrogradely connected neurons *in vivo.* Inputs to choline acetyltransferase (ChAT)-expressing neurons in the striatum were used for this test. These neurons primarily receive input from the cortex and the thalamus (Thomas et al., 2000; Bloomfield et al., 2007) (Figure 7A). In order to mark this population, we crossed ChAT-Cre mice to Ai9 mice, which express tdTomato in cells with a Cre expression history (Madisen et al., 2010) Six-week-old mice from this cross were injected in the CP with two AAV vectors: one expressing a Cre-conditional (“floxed”) TVA-mCherry fusion protein, and another expressing a floxed RABV-G. Two weeks later, the mice were injected in the same coordinates with rVSVΔG with the EnvA/RABV-G chimeric glycoprotein on the virion surface [rVSVΔG(A/RG)] (Beier et al., 2011). Cells successfully infected with these two AAV vectors could host infection by a rVSV and should be able to produce rVSV virions with RABV-G on the surface. Such starter cells should also express tdTomato and GFP. If rVSV were to be produced, and if it were to transmit across the synapse retrogradely, cortical and thalamic neurons should be labeled by GFP.

Mice injected with these AAV and rVSV viruses were sacrificed 5 days after rVSV infection, and brains analyzed for fluorescence. As expected for starter cells, some neurons in the CP expressed both tdTomato and GFP (Figure 7B). Outside of the CP, small numbers of GFP+ neurons that were not mCherry+ were observed in the cortex (Figures 7C,D) and thalamus (Figure 7E), consistent with retrograde spread. Control animals not expressing Cre, or not injected with AAV encoding RABV-G, did not label cells in the cortex or thalamus (*N* = 3 for both controls and experimental condition).

## Discussion

### rVSV(RABV-G) is a retrograde tracer in the CNS

Here, we report on the use of rVSV as a retrograde transsynaptic tracer for CNS circuitry. VSV can be modified to encode the RABV-G protein in the viral genome, allowing the virus to replicate and transmit across multiple synaptically connected cells, i.e., as a polysynaptic tracer. Alternatively, if the virus has the G gene deleted from its genome and RABV-G is provided *in trans*, it behaves as a monosynaptic tracer (Beier et al., 2011). Although it has been known for many years that RABV travels retrogradely among neurons (Astic et al., 1993; Ugolini, 1995; Kelly and Strick, 2003), and pseudotyping lentiviruses with RABV-G is sufficient for axonal transport (Mazarakis et al., 2001), the retrograde transmission specificity among neurons had not been clearly shown to be a property of the G protein itself, as it might have been due to other viral proteins in addition to, or instead of, the viral G protein. Since native VSV does not have these retrograde transsynaptic properties (van den Pol et al., 2002; Beier et al., 2011), and the only alteration to the VSV genome was the substitution of the VSV G gene with the G gene of RABV, it is clear that the RABV glycoprotein is responsible for retrograde direction of viral transmission across synapses, at least in the case of rVSV.

### VSV as a viral vector for the CNS: rapid gene expression and genome capacity

The early onset of gene expression from VSV relative to RABV (one hour vs. multiple hours) makes it beneficial in experimental paradigms in which the experiment needs to be done within a narrow window of time, such as tissue slices and explants. In addition, more than one transgene can be encoded in the viral genome without the need of a 2A or IRES element. The use of the first position of the genome enhances the expression level of the transgene inserted at that location, since VSV (and RABV) express genes in a transcriptional gradient; therefore, the first gene is the most highly transcribed (Knipe, 2007). This leads to rational predictions of expression levels so that one can choose the position of insertion of a transgene, or transgenes, according to this gradient and the desired level of expression. The size of the viral capsid is apparently not rigid, allowing for the inclusion of genomes that are substantially larger than the native genome, unlike the rigid capacity for some other viral vectors, such as AAV (Duan et al., 2000; Yan et al., 2000).

### Sufficiency of glycoproteins to confer directionality of rvsv spread enables novel applications

The fact that VSV can be made to spread anterogradely (Beier et al., 2011) or retrogradely across synapses with the change of a single gene affords several advantages over viral tracers that heretofore have not shown such flexibility in the directionality of tracing. In addition to the obvious application of tracing anterograde connections, combinations can be made to exploit the different forms of the virus. One example that employs the simultaneous infection with an anterograde and retrograde form of VSV is demonstrated in Figure 6. This experiment was designed to address whether the anterograde projections from the cortex to the CP would label the same brain regions as were labeled by a retrograde virus injected into the SN. Although a block of superinfection by the virus may preclude infection of the same cell with multiple rVSVs, adjacent cells could still become labeled by different viruses (Whitaker-Dowling et al., 1983). The observed results could be due to a preferential labeling by the anterograde transsynaptic virus of indirect pathway MSNs in this experiment, which then synapse onto the GP, thereby reflecting a viral bias. Alternatively, it could indicate that the cortical neurons in the injected region largely do not label the MSNs that project to the area of the SN injected with the retrograde virus. One further possibility is that too little virus was used to observe co-labeling of a given region. However, given the density of infection (i.e., Figures 6I,J), the latter possibility seems unlikely. Additionally, the spread of the polysynaptic rVSV(RABV-G) appears to attenuate with increasing numbers of synapses crossed, permitting an analysis of more restricted viral spread. This is quite fortuitous, as if spread were to continue, it would lead to widespread infection and lethality. In addition, reconstruction of connectivity would be more difficult. This reduced efficiency appears to also hold for the monosynaptic form of VSV complemented with RABV-G, as the efficiency of transmission appeared lower than the comparable experiment with RABV (Watabe-Uchida et al., 2012). This is likely due to viral attenuation when VSV-G is replaced with RABV-G.

### Advantages of VSV over other viral tracers: safety

We were attracted to the use of VSV as a viral tracer due to its long track record as a safe, replication-competent laboratory agent. Laboratory workers using VSV have not contracted any diseases, and natural VSV infections among human populations in Central America and the southwestern United States (Rodríguez, 2002) occur without evident pathology (Johnson et al., 1966; Brody et al., 1967). VSV was thus an attractive candidate for its use as a polysynaptic tracer for CNS studies, which requires an ability to replicate through multiple transmission cycles. Both replication-competent and incompetent forms of VSV are in use under Biosafety Level 2 containment. Replication-competent RABV is Biosafety Level 3, due to the fact that infection with replication-competent RABV is almost always fatal to humans and in mice when infected intracerebrally (Smith, 1981; Knipe, 2007).

Differences in pathogenicity between VSV and RABV are likely due to the ability of RABV to evade the innate immune system, particularly interferon (Hangartner et al., 2006; Junt et al., 2007; Lyles and Rupprecht, 2007; Rieder and Conzelmann, 2009; Iannacone et al., 2010). VSV infection efficiently triggers an interferon response, and it has not evolved a method of escape from this response, unlike RABV (Brzózka et al., 2006). In fact, VSV is being pursued as a vaccine for other viruses, including RABV (Lichty et al., 2004; Publicover et al., 2004; Kapadia et al., 2005; Schwartz et al., 2007; Iyer et al., 2009; Geisbert and Feldmann, 2011). VSV does not typically spread beyond the initially infected site in the periphery (Kramer et al., 1983; Vogel and Fertsch, 1987). This likely is the cause of the minor or absent symptoms in humans and animals infected in nature. Polysynaptic VSV vectors are thus predicted to be much safer than polysynaptic RABV vectors. We have tested this prediction by injecting a series of mice in the footpads and hind leg muscles with rVSV(RABV-G), with the result that no injected animals showed any evidence of morbidity or mortality (Beier, Goz et al., in preparation).

While safer for laboratory workers than RABV, the main drawback to using VSV is its rapid cellular toxicity (van den Pol et al., 2009; Beier et al., 2011). Toxicity is due to suppression of cellular transcription and a block in the export of cellular RNAs from the nucleus to the cytoplasm (Black and Lyles, 1992; Her et al., 1997; Ahmed and Lyles, 1998; Petersen et al., 2000; von Kobbe et al., 2000), as well as inhibition of the translation of cellular mRNAs (Francoeur et al., 1987; Jayakar et al., 2000; Kopecky et al., 2001). VSV is much quicker to enact its gene expression program than is RABV, such that cells suffer the toxic effects more quickly than after RABV infection. One aspect of VSV that can be exploited in the future to ameliorate the speed of toxicity is the use of VSV mutants and variants. One such mutant is the M51R, which permitted us to conduct physiological analyses of pre-and post-synaptic cells (Beier et al., 2011). We are in the process of examining the transmission properties of this mutant *in vivo*, as well as the effects of other mutations or viral variants on prolonging the health of neurons after infection.

## Summary

rVSV vectors can be used to study the connectivity of neuronal circuitry. In addition to combinations of replication-competent forms of VSV, the replication-incompetent, monosynaptic forms of the virus can be easily combined, without the need to change viruses (Beier et al., 2011). This allows a straightforward way to study both the projections into, and out from, a genetically defined cell population. This can be done with the same viral genome, with the only change needed being the glycoprotein, for the selection of the direction of transmission. This flexibility of VSV makes it a powerful, multi-application vector for studying connectivity in the CNS.

## Materials and methods

### Virus construction and production

All rVSV clones were cloned from the rVSVΔG backbone (Chandran et al., 2005). mCherry, Kusabira orange, Venus, and CFP were cloned into the first (GFP) position using XhoI and MscI sites, and VSV-G (a gift from Richard Mulligan, Harvard Medical School, Boston, MA) and RABV-G (a gift from Ed Callaway, Salk Institute, San Diego, CA) were cloned into the fifth (G) position using the MluI and NotI restriction sites. Genes for fluorescent proteins were obtained from Clontech.

Viruses were rescued as previously described (Whelan et al., 1995). At 95% confluency, eight 10 cm plates of BSR cells were infected at an MOI of 0.01. Viral supernatants were collected at 24-h time intervals and ultracentrifuged at 21,000 RPM using a SW28 rotor and resuspended in 0.2% of the original volume. For titering, concentrated viral stocks were applied in a dilution series to 100% confluent BSR cells and plates were examined at 12 hpi. Viral stocks were stored at −80°C.

For ΔG viruses, 293T cells were transfected with PEI (Ehrhardt et al., 2006) at 70% confluency on 10 cm dishes with 5 μg of pCAG-RABV-G. Twenty-four hours post-infection, the cells were infected at an MOI of 0.01 with rVSVΔG expressing either GFP or mCherry. Viral supernatants were collected for the subsequent 4 days at 24 h intervals.

Virus preparations are now available from the Salk GT3 viral core (http://vectorcore.salk.edu/). All plasmids are available from Addgene (http://www.addgene.org/).

### AAV vectors

AAV-FLEx-RABV-G and AAV-FLEx-TVA-mCherry plasmids originated from the Lab of Naoshige Uchida (Watabe-Uchida et al., 2012), and virus stocks were generous gifts from Brad Lowell, Harvard Medical School.

### Injections of mice

ChAT-Cre (B6;129S6-Chat^tm1(cre)Lowl^/J) and Ai9 (B6.Cg-Gt(ROSA)26Sor<tm9(CAG-tdTomato)Hze>/J) mice were obtained from the Jackson Laboratory (Madisen et al., 2010).

Eight-week-old CD-1 mice were injected using pulled capillary microdispensers (Drummond Scientific, Cat. No: 5-000-2005), using coordinates from The Mouse Brain in Stereotaxic Coordinates (Franklin and Paxinos, 1997). Injection coordinates (in mm) used were:

Primary Motor Cortex: A/P +1.34 from bregma, L/M 1.7, D/V −1 from pial surface
LGN: A/P −2.46 from bregma, L/M 2, D/V −2.75
Superior Colliculus: A/P −3.88 from bregma, L/M 0.5, D/V −1
CP: A/P +1 from bregma, L/M 1.8, D/V −2.5
Primary Visual Cortex (V1): A/P −3.4 from bregma, L/M 2.5, D/V −0.8.
SNr: A/P −3.28 from bregma, L/M 1.5, D/V −4.25

For multi-color analysis (Figures 1C,D), 3 × 10^9^ ffu/mL rVSV was injected into various regions. For CP injections, 100 nL of rVSV(RABV-G) or rVSV(VSV-G) at 3 × 10^7^ ffu/mL was injected at a rate of 100 nL/min. For the replication-incompetent viruses, 100 nL of 1 × 10^7^ ffu/mL rVSVΔG(RABV-G) or rVSVΔG (VSV-G) was injected. In the motor cortex, 100 nL of 1 × 10^7^ ffu/mL rVSV(RABV-G) was injected, and mice harvested 2 dpi. For V1 injections, 100 nL of 3 × 10^10^ ffu/mL rVSV(RABV-G) was injected, and mice were examined 3 or 7 dpi.

For infections of the dura mater, 1 μL of 3 × 10^10^ ffu/mL rVSV(RABV-G) was applied to the surface of the dura. The virus was allowed to absorb, and the surface was subsequently covered in bone wax, and the wound sutured.

For co-injections of virus into the same animal, 100 nL of a combination of 3 × 10^7^ ffu/mL rVSV(VSV-G) and 3 × 10^8^ ffu/mL rVSV(RABV-G) were co-injected into the motor cortex, and brains examined 3 dpi. For injections of the viruses into different regions, 100 nL of 3 × 10^7^ ffu/mL rVSV(VSV-G) was injected into M1, and 100 nL of 3 × 10^8^ ffu/mL rVSV(RABV-G) into the SNr, and brains examined 3 dpi. A lower titer of rVSV(VSV-G) was used, as rVSV(RABV-G) is attenuated.

All mouse work was conducted in biosafety containment level 2 conditions and was approved by the Longwood Medical Area Institutional Animal Care and Use Committee.

### Slice preparation and pharmacology

Recordings were made from cortical pyramidal neurons in slices taken from postnatal day 12–18 mice, inoculated in the CP 12–18 h prior with rVSV(RABV-G). Coronal slices (300 μm thick) were cut in ice-cold external solution containing (in mM): 110 choline, 25 NaHCO_3_, 1.25 NaH_2_PO_4_, 2.5 KCl, 7 MgCl_2_, 0.5 CaCl_2_, 25 glucose, 11.6 Na-ascorbate, and 3.1 Na-pyruvate, bubbled with 95% O_2_ and 5% CO_2_. Slices were then transferred to artificial cerebrospinal fluid (ACSF) containing (in mM): 127 NaCl, 25 NaHCO_3_, 1.25 NaH_2_PO_4_, 2.5 KCl, 1 MgCl_2_, 2 CaCl_2_, and 25 glucose, bubbled with 95% O_2_ and 5% CO_2_. After an incubation period of 30–40 min at 34°C, slices were stored at room temperature. All experiments were conducted at room temperature (25°C). In all experiments, 50 μM picrotoxin, 10 μM 2,3-Dioxo-6-nitro-1,2,3,4-tetrahydrobenzo[f]quinoxaline-7-sulfonamide (NBQX), and 10 μM 3-((R)-2-Carboxypiperazin-4-yl)-propyl-1-phosphonic acid (CPP) were present in the ACSF to block GABAA/C, AMPA, and NMDA receptor-mediated transmission, respectively. All chemicals were from Sigma or Tocris.

### Electrophysiology and imaging

Whole-cell recordings were obtained from infected and uninfected deep layer cortical pyramidal neurons identified with video-IR/DIC and GFP fluorescence was detected using epifluorescence illumination. With the deep layers of the cortex, 2-photon laser scanning microscopy (2PLSM) was used to confirm the cell types based on morphology. Deep layer pyramidal neurons had large cell bodies, classic pyramidal shape and dendritic spines. Glass electrodes (2–4 MΩ) were filled with internal solution containing (in mM): 135 KMeSO_4_, 5 KCl, 5 HEPES, 4 MgATP, 0.3 NaGTP, 10 Na_2_HPO_4_, 1 EGTA, and 0.01 Alexa Fluor-594 (to image neuronal morphology) adjusted to pH 7.4 with KOH. Current and voltage recordings were made at room temperature using a AxoPatch 200B or a Multiclamp 700B amplifier. Data was filtered at 5 kHz and digitized at 10 kHz.

### Data acquisition and analysis

Imaging and physiology data were acquired and analyzed as described previously (Carter and Sabatini, 2004). Resting membrane potential was determined by the average of three 5-s sweeps with no injected current. Passive properties of the cell, membrane (Rm) and series resistance (Rs) and capacitance (Cm), were measured while clamping cells at −65 mV and applying voltage steps from −55 to −75 mV. The current—firing relationship was determined in current clamp with 1-s periods of injected current from 100 to 500 pA.

### Hippocampal slice cultures

The time course of viral gene expression experiments were carried out in organotypic hippocampal slice cultures prepared from postnatal day 5–7 Sprague-Dawley rats as described previously (Stoppini et al., 1991). Slices were infected after 7 days *in vitro*, and images were acquired on a two-photon microscope.

#### Conflict of interest statement

The authors declare that the research was conducted in the absence of any commercial or financial relationships that could be construed as a potential conflict of interest.
